# Rheumatic Fever Follow-Up Study (RhFFUS) protocol: a cohort study investigating the significance of minor echocardiographic abnormalities in Aboriginal Australian and Torres Strait Islander children

**DOI:** 10.1186/1471-2261-12-111

**Published:** 2012-11-27

**Authors:** Marc Gerard Wootton Rémond, David Atkinson, Andrew White, Yvonne Hodder, Alex DH Brown, Jonathan R Carapetis, Graeme Paul Maguire

**Affiliations:** 1 School of Medicine and Dentistry, Faculty of Medicine, Health and Molecular Sciences, James Cook University, Cairns, QLD, Australia; 2 Rural Clinical School of Western Australia, The University of Western Australia, Broome, WA, Australia; 3 Kimberley Aboriginal Medical Services Council, Broome, WA, Australia; 4 School of Medicine and Dentistry, James Cook University, Townsville, QLD, Australia; 5 South Australian Health and Medical Research Institute, Adelaide, SA, Australia; 6 Telethon Institute for Child Health Research, Centre for Child Health Research, University of Western Australia, Perth, WA, Australia; 7 Baker IDI, Alice Springs, Northern Territory, Australia

**Keywords:** Rheumatic heart disease, Acute rheumatic fever, Screening, Aboriginal, Torres Strait Islander, Indigenous, Diagnosis, Prevention, Australia, Echocardiography

## Abstract

**Background:**

In Australia, rheumatic heart disease (RHD) is almost exclusively restricted to Aboriginal Australian and Torres Strait Islander people with children being at highest risk. International criteria for echocardiographic diagnosis of RHD have been developed but the significance of minor heart valve abnormalities which do not reach these criteria remains unclear. The Rheumatic Fever Follow-Up Study (RhFFUS) aims to clarify this question in children and adolescents at high risk of RHD.

**Methods/design:**

RhFFUS is a cohort study of Aboriginal and/or Torres Strait Islander children and adolescents aged 8–17 years residing in 32 remote Australian communities. Cases are people with non-specific heart valve abnormalities detected on prior screening echocardiography. Controls (two per case) are age, gender, community and ethnicity-matched to cases and had a prior normal screening echocardiogram. Participants will have echocardiography about 3 years after initial screening echocardiogram and enhanced surveillance for any history suggestive of acute rheumatic fever (ARF). It will then be determined if cases are at higher risk of (1) ARF or (2) developing progressive echocardiography-detected valve changes consistent with RHD.

The occurrence and timing of episodes of ARF will be assessed retrospectively for 5 years from the time of the RhFFUS echocardiogram. Episodes of ARF will be identified through regional surveillance and notification databases, carer/subject interviews, primary healthcare history reviews, and hospital separation diagnoses.

Progression of valvular abnormalities will be assessed prospectively using transthoracic echocardiography and standardized operating and reporting procedures. Progression of valve lesions will be determined by specialist cardiologist readers who will assess the initial screening and subsequent RhFFUS screening echocardiogram for each participant. The readers will be blinded to the initial assessment and temporal order of the two echocardiograms.

**Discussion:**

RhFFUS will determine if subtle changes on echocardiography represent the earliest changes of RHD or mere variations of normal heart anatomy. In turn it will inform criteria to be used in determining whether secondary antibiotic prophylaxis should be utilized in individuals with no clear history of ARF and minor abnormalities on echocardiography. RhFFUS will also inform the ongoing debate regarding the potential role of screening echocardiography for the detection of RHD in this setting.

## Background

Acute rheumatic fever (ARF) is an auto-immune condition resulting from infection with group-A streptococcus (GAS) [1,2]. In susceptible individuals, two to three weeks following throat [3,4] and possibly skin [5] infection, a delayed inflammatory response associated with ARF can occur directed at the heart (carditis), brain (chorea), joints (arthralgia/arthritis) and skin (rash). While the effects of ARF are usually transitory, recurrent episodes of ARF-related carditis can result in long-term damage to heart valves called rheumatic heart disease (RHD) [6,7]. The mitral and aortic valves are particularly prone to this damage.

In Australia, acquisition of ARF and RHD is now almost exclusively restricted to the local indigenous population comprising Aboriginal Australians and/or Torres Strait Islander peoples. This is particularly the case for those living in regional and remote areas of central and northern Australia [8]. Rates of ARF and RHD in these regions are among the highest documented in the world [9]. RHD results in significant morbidity [10] and health care utilization [10] and can lead to premature death [8].

Whilst the long-term priority for addressing ARF and RHD remains identifying effective targets for primary prevention, to date these have proven elusive [1,11]. Hence, the current emphasis remains the secondary prevention of GAS infection with prophylactic antibiotics in people with a history of ARF or known RHD [1,12,13]. This has been demonstrated to prevent recurrent ARF and progression of RHD [14-17].

Central to the delivery of ARF/RHD secondary prevention programs is the diagnosis of ARF and RHD. A diagnosis of ARF is classically made in accordance with the Jones criteria based on a combination of major and minor symptoms, signs and investigation results [1,18,19]. Nonetheless, given the variable manifestations of ARF, cases can be missed [20]. In high-risk populations in Australia, the Jones criteria have been modified with the aim of improving their sensitivity [1].

Prior to the introduction of echocardiography (heart ultrasound), diagnosis of RHD required an experienced clinician with the requisite skill to identify and correctly interpret findings detected on auscultation of the heart. Nonetheless it has been shown auscultation alone is neither sensitive [21] nor specific [22]. The increasing availability of more portable and affordable echocardiography to assess heart valve morphology and function (stenosis or regurgitation) has resulted in significant debate regarding the diagnosis of RHD on echocardiography alone. This particularly relates to the ability of echocardiography to confirm or refute a diagnosis of RHD. In an attempt to address this issue, the World Heart Federation (WHF) recently released criteria [23] for the diagnosis of RHD based on both morphological and functional findings on echocardiography. The WHF criteria include a category of “Borderline” RHD, recognizing potential abnormalities on echocardiography that are of uncertain significance.

The importance of such minor abnormalities was highlighted by a recent Australian RHD prevalence and echocardiography validation study. The gECHO (getting Every Child’s Heart Okay) Study undertook echocardiographic screening of 3978 high risk (Aboriginal Australian and/or Torres Strait Islander) and 1267 low risk (non-Indigenous Australian) children across northern and central Australia. Preliminary results revealed a number of children with mild potential abnormalities of doubtful significance (personal communication Graeme Maguire). If these abnormalities are representative of the earliest changes of RHD then offering such children regular secondary prophylaxis may prevent disease progression. This question has been identified as a priority for future investigation [24,25].

The Rheumatic Fever Follow-Up Study (RhFFUS) aims to clarify the significance of minor echocardiographic abnormalities in children and adolescents at high risk of ARF/RHD. More specifically, it aims to determine if Aboriginal and Torres Strait Islander children and adolescents aged 8–17 years with a previous potentially abnormal but non-diagnostic screening echocardiogram are at higher risk of (1) contracting ARF or (2) progressive echocardiography-detected changes consistent with RHD.

### Hypothesis

Children from a population at increased risk of ARF and RHD who have minor and non-specific heart valve abnormalities on screening echocardiography are more likely to:

• have subsequent episodes of ARF and/or

• develop progressive echocardiographic changes consistent with RHD

than age, gender, ethnicity and community-matched children who had a previously normal echocardiogram.

## Methods/design

### Study design

**Figure 1:**
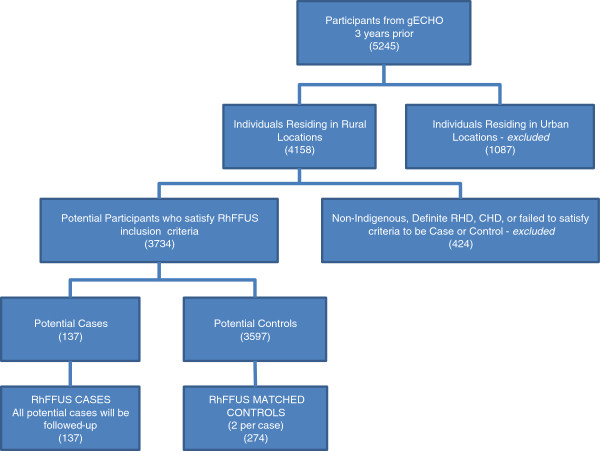
**Protocol for selection of participants for RhFFUS.** “Rural locations” comprise those communities involved in the gECHO that are outside of the cities of Cairns and Darwin. “Urban locations” comprise communities involved in the gECHO study that are within the cities of Cairns and Darwin. “RhFFUS inclusion criteria” are outlined in the text. “Non-Indigenous” refers to subjects enrolled in the gECHO project who self-reported as not being Aboriginal and/or Torres Strait Islander. “Definite RHD” is defined by WHF criteria [23]. “CHD” refers to a diagnosis of congenital valvular heart disease that may generate morphologic or functional abnormalities similar to RHD (bicuspid aortic valve, dilated aortic root, mitral valve prolapse). “Case or Control” criteria are defined in the text.

RhFFUS is a cohort study of children with non-specific mitral and/or aortic valve abnormalities detected on prior screening echocardiography. Children will be assessed prospectively for the development of progressive valve abnormalities and retrospectively for the incidence of ARF. The comparator will be age, gender, community and ethnicity-matched controls who have previously had a normal screening echocardiogram (see Figure 1).

### Study populations

**Figure 2:**
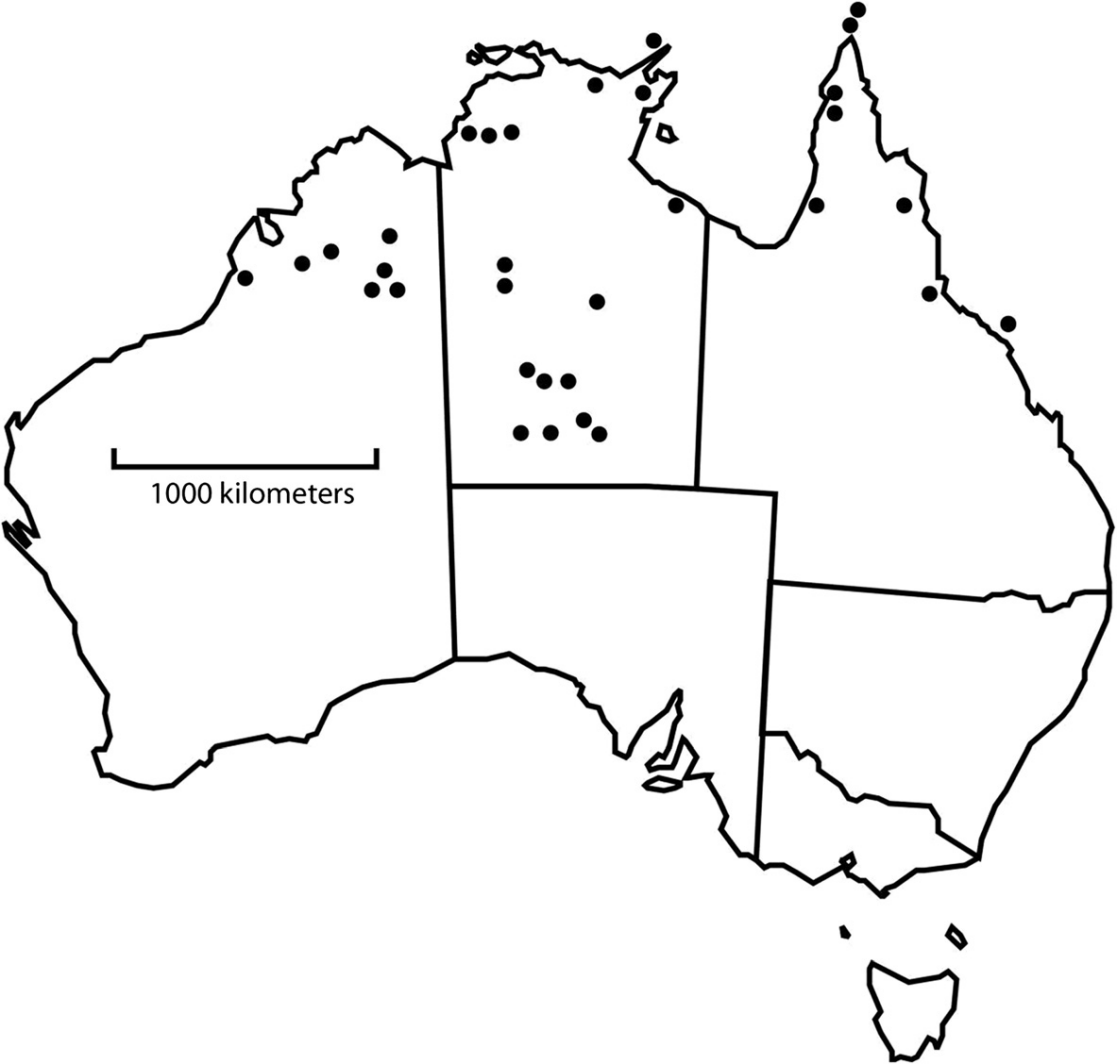
The 32 RhFFUS sites from Western Australia, the Northern Territory, and far north Queensland.

Participants in this study will comprise a subset of children who had an echocardiogram as part of the earlier gECHO screening study. These participants reside in 32 remote communities across northern and central Australia (see Figure 2).

### Inclusion criteria

Children who participated in the gECHO study will be eligible for inclusion in RhFFUS if they identify as Aboriginal Australian and/or Torres Strait Islander and live in a remote location.

### Exclusion criteria

Children who participated in the gECHO study will not be eligible for inclusion in RhFFUS if they identify as non-Indigenous Australians, live in urban locations, had a diagnosis of definite RHD based on gECHO screening, or had a diagnosis of congenital valvular heart disease that may generate morphologic or functional abnormalities similar to RHD (bicuspid aortic valve, dilated aortic root, mitral valve prolapse).

### Case/control definition

Cases will be children with non-specific findings on their gECHO screening echocardiogram. More specifically, their screening echocardiogram from gECHO must not fulfill WHF echocardiography criteria for definite RHD [23] and must meet one of the echocardiographic criteria outlined below. Given the uncertainty of interpretation of minor echocardiographic abnormalities, criteria for cases are more sensitive than WHF criteria for borderline RHD [23]; 47 of the 137 cases included in RhFFUS satisfy the criteria for borderline RHD. Controls will be children who had a normal echocardiogram as outlined below.

Cases:

1. One or more morphologic changes of the mitral valve (MV)* and/or aortic valve (AV)* without pathologic mitral regurgitation (MR)* or aortic regurgitation (AR)*; or

2. Pathologic MR* with no or one morphologic feature of RHD* or pathologic AR* with no or one morphologic feature of RHD* (not both); or

3. Multiple MR jets and/or multiple AR jets (in at least two views) that do not fulfill criteria for pathologic MR* or AR*

Note: *Criteria for morphologic changes of MV and AV, and pathologic AR and MR as described by WHF criteria [23].

Controls:

1. No morphologic features of RHD of the AV or MV; and

2. MR < 1 cm; and

3. No AR; and

4. No other acquired or congenital valvular heart disease

Note: *Criteria for morphologic changes of MV and AV, and pathologic AR and MR as described by WHF criteria [23].

The matching process will involve stratifying all eligible gECHO participants by community, gender and age. For each identified case, the closest age-matched control from the same community and of the same gender will then be selected and assigned to the case. This process will be repeated so that two matched controls are assigned to each case.

### Sample size

Sample size estimations are based on projected rates of ARF based on the annual incidence of ARF in people with known RHD (2.5%) and the background annual incidence of ARF in 5 to 14 year old Aboriginal and Torres Strait Islander children in the NT (0.27%) (personal communication Northern Territory ARF/RHD register) over a five year period. Based on an assumed alpha of 0.05 and beta of 0.1 (power of 90%) detecting a difference of one or more episodes of ARF in 12.5% of cases and 1.35% of controls over five years of follow-up, and using a ratio of cases to controls of 1:2, would require a sample size of 83 cases and 165 controls or a total number of 248 reviews. While it is apparent that carditis in the acute setting of ARF may resolve in up to half of cases [26] there are no clear data to inform the powering of progression of non-specific echocardiographic changes. Nonetheless, if it is assumed at most 5% of controls will develop morphologic and function valvular changes on echocardiography compared with 20% of those with pre-existing non-specific changes then it would require the follow-up of 77 cases and 154 controls to detect such a difference with the tolerances above, a number of enrolments well within the projected subject numbers.

### Informed consent

Parents, carers or guardians of all identified subjects will be informed regarding the study using local Indigenous research staff and local language translators as required. Written informed consent will be obtained. In addition written assent will be obtained from subjects who are 14 years and older. Subjects who are 16 years and older, who fulfill the criteria for mature minors [27] and who are not living with the people who would normally be identified as parents, carers or guardians will be able to provide their own consent.

### Enrolling participants

Potential participants will be approached with the assistance of local research assistants and the local primary health care centre. In addition, RhFFUS staff will visit the homes of potential participants in the company of a local health care staff member or a community-based Indigenous Australian research assistant in order to contact and enroll potential participants. If potential cases have moved to another location since the gECHO project, attempts will be made to ascertain their most recent address and, if feasible, to contact and follow them up there.

### Data collection and outcomes

Outcome data for cases and controls will include ARF incidence and the development or progression of mitral and/or aortic valve abnormalities.

(a) ARF incidence

The occurrence and timing of episodes of ARF will be assessed retrospectively for 5 years from the time of the RhFFUS screening echocardiography. Anecdotal evidence suggests that in the setting where RhFFUS is being conducted, episodes of ARF may be missed or not recorded owing to insufficient diagnostic information being collected at time of presentation to primary health care sites. Thus, in order to increase the power of RhFFUS to detect differences between case and controls, four categories of ARF will be used as outcome variables (see Table 1).

**Table 1:** Criteria for ARF, definite, probable, possible, potential

**Diagnosis**	**Criteria**
Definite ARF	Australian modified Jones criteria for high risk populations (includes echocardiographic evidence of carditis and monoarthritis as major criteria) [1]
Probable ARF	•Arthritis/arthralgia; and
	•One or more of: temperature ≥ 38°C, C-reactive protein ≥ 30 mg/L, erythrocyte sedimentation rate ≥ 30 mm/h, prolonged P-R interval on ECG*; and
	•GAS infection**; and
	•No other diagnosis [28]
Possible ARF	•Arthritis/arthralgia; and
	•GAS infection**; and
	•No other diagnosis
Potential ARF	•Arthritis/athralgia; and
	•No other diagnosis

*Upper limits of normal of P-R interval are: 3–12 years, 0.16s; 12–16 years, 0.18s; 17+ years, 0.20s [1].

**GAS infection is defined as throat swab positive for GAS on culture or serology consistent with recent GAS infection including elevated antistreptolysin O titre and antideoxyribonuclease B antibodies as outlined in the Australian guideline for prevention, diagnosis and management of acute rheumatic fever and rheumatic heart disease (2nd edition) [1].

To gain a comprehensive overview of ARF episodes a number of sources of data will be examined. These comprise: regional surveillance and notification databases; carer/subject interviews; primary healthcare history reviews; and hospital separation diagnoses.

At time of enrolment an interview of the participants and/or their carers/family will be undertaken. Data collected will comprise: demographics, knowledge of ARF or RHD diagnoses, episodes suggestive of ARF (arthritis/arthralgia), whether the participant is receiving secondary prophylaxis, household crowding and socioeconomic data.

A primary health care and hospital history review will also be undertaken in a representative subset of participants. Data collected during this review will comprise information about potential episodes of ARF, arthritis/arthralgia, chorea and diagnoses of RHD. Clinical data regarding each potential episode of ARF, based on the Australian modified Jones criteria [1], will be collected. These data will be used to validate the accuracy of existing register-based ARF notifications.

(b) Progression of valvular abnormalities

Progression of valvular abnormalities will be assessed prospectively using transthoracic echocardiography and standardized operating and reporting procedures already developed and refined for the gECHO study. Briefly the echocardiogram will be undertaken using a Vividi/e portable cardiac ultrasound machine (GE Healthcare) with a standardized machine setup as outlined below.

• Highest frequency transducer that gives adequate penetration,

• Colour gain set by gradually increasing until static background noise barely appears,

• No electrocardiography (ECG) for screening studies,

• ECG for comprehensive studies,

• At least 2 second video acquisition of each view with longer periods for detailed sweeping of potentially abnormal valves

Studies will initially involve a screening echocardiogram which will proceed to a comprehensive study if there is one or more of: mitral regurgitation ≥ 10mm; any aortic regurgitation; any other abnormal mitral or aortic valve findings; or any other pathology present (e.g. abnormal morphology, thickening, multiple regurgitant jets etc.).

**Table 2:** View and assessments required for RhFFUS screening echocardiogram

**Echocardiographic view**	**Assessment**
Parasternal long axis (PLAX) 2 dimensional (2D)	Mitral and aortic valves to assess the morphology of these valves
	AMVL and posterior mitral valve leaflet (PMVL) thickness
	AMVL - ensure view is on axis and measure the thickest point of AMVL in late diastole when AMVL parallel with the IVS
	PMVL - ensure view is on axis and measure the thickest point of the PMVL mid diastole, exclude chordae from the measurements.
PLAX colour Doppler	Colour Doppler to view the mitral and aortic valves for evidence of regurgitation, include lateral and inferior sweeps
Parasternal short axis (PSAX) 2D	View of the mitral and aortic valves to assess morphology
PSAX colour Doppler	Colour Doppler to view the mitral and aortic valves for evidence of regurgitation, include lateral and inferior sweeps
Apical 2D	Apical 4/5 chamber view of mitral and aortic valves for morphology
Apical colour Doppler	Colour Doppler to view the mitral and aortic valves for evidence of regurgitation

Screening echocardiograms will incorporate the views and assessments outlined in Table 2. Comprehensive echocardiograms, if required, will incorporate more detailed information based on the abnormalities detected on the screening study and will be undertaken using the views and assessments outlined in Table 3. All echocardiograms will be carried out by trained, accredited and practicing echocardiographers.

**Table 3:** Requirement, view and assessments required for RhFFUS comprehensive echocardiogram

**Potential abnormality**	**View and Assessment**
All comprehensive studies	PLAX 2D assessment of:
	- left ventricular chamber dimensions at the level of the mitral valve leaflet tips (interventricular septum thickness, left ventricular end-diastolic & systolic dimensions, left ventricular posterior wall thickness)
	- aorta and left atrium diameter at the aortic cusp level
Dependent on the presence of potential mitral or aortic valve disease	Standardized additional studies including routine acquisition of colour, continuous and pulse wave Doppler measurements
Any other pathology	As per routine clinical protocols

Progression of a valve lesion will be defined as one or more of: development of any morphologic or function abnormality in a control subject; development of a new functional or morphologic abnormality in a case; or progression of severity of a functional valve lesion (regurgitation/stenosis) based on standard severity criteria [29].

Progression of valve lesions will be determined by specialist cardiologist readers who will be provided with the initial gECHO screening and subsequent RhFFUS screening echocardiogram for each participant. Studies will be read and assessed individually and then in pairs. The readers will be blinded to the initial gECHO assessment and temporal order of the two echocardiograms. Reporting will use a standardized reporting template.

### Blinding

In order to limit any potential for information bias in this study only the study coordinator at each site will know whether a participant is a case or control. Thus the sonographer carrying out the echocardiogram, the researchers involved in reviewing each participant’s medical history and the cardiologist assessing the echocardiogram will be blinded to the participant’s status as case or control.

### Data management

All data collected on paper-based forms (participant/carer/family interviews and medical history reviews) will be stored under numerical code in a locked filing cabinet in the RhFFUS study coordinator’s office. Only research personnel will have access to these records.

Reports from the echocardiogram readers will be received in electronic format and will be saved in a password-protected folder on the study coordinator’s computer.

Research staff will transfer the information from both paper forms and electronic echocardiogram reports to an Access database (Microsoft Office Access 2007, Microsoft Corporation, Redmond, Washington, USA) that will be password-protected. De-identified data will be analysed using STATA version 12 (StataCorp, College Station, Tex, USA).

Any information collected will be strictly confidential and no identifying information will be published or disseminated upon completion of the study. Data will be stored for at least 5 years as per Australian National Health and Medical Research Council guidelines [30].

### Statistical analysis

The primary analysis will be based on univariate analysis comparing cases and matched controls. This will include a χ^2^ analysis comparing the number of children with an episode of ARF (stratified by definite, probable, possible and potential (see Table 1)) during the period of follow-up and those who have demonstrated progression of a valve lesion (see above). More detailed analysis will include survival analysis comparing the timing of first episode of ARF to address potential loss of follow-up and Poisson regression for the rate of ARF to address the potential occurrence of more than one episode of ARF occurring in any one individual and a variable period of follow-up. Multivariate techniques (logistic regression and Cox proportional hazard) will be utilized if the matching of cases and controls is not successful and to addresses subsequently identified covariates including the possibility of concomitant prophylactic antibiotics.

### Ethics

RhFFUS has been approved by human ethics research committees in each of the jurisdictions where it will be undertaken. Approval has been granted by the following committees: Darling Downs – West Moreton (Toowoomba and Darling Downs) Health Service District Human Research Ethics Committee (Queensland)(HREC/11/QTDD/10), James Cook University Human Research Ethics Committee (Queensland)(H4136), Central Australian Human Research Ethics Committee (Northern Territory)(HREC-12-35), the Human Research Ethics Committee of Northern Territory department of Health and Menzies School of Health Research (Northern Territory)(HREC-2011-1564), the WA Country Health Service Research Ethics Committee (Western Australia)(2011:31), the Western Australian Aboriginal Health Ethics Committee (Western Australia)(371-10/11), the University of Western Australia Human Research Ethics Committee (Western Australia)(RA/4/1/5313).

### Funding

The RhFFUS project is funded by the Australian Government through a grant from the National Health and Medical Research Council (NHMRC Project Grant Application 1005951).

## Discussion

The results of RhFFUS will be integral in informing the future response to ARF and RHD in Australia. In particular, RhFFUS will clarify the criteria to be used in determining whether secondary antibiotic prophylaxis should be prescribed in individuals with no clear history of ARF but minor potential abnormalities detected on echocardiography suggestive, but not diagnostic, of RHD. RhFFUS will also help inform the ongoing debate regarding the potential role of screening echocardiography in this setting. In particular, it will allow clinicians to understand the significance of subtle changes on echocardiography and to determine whether these represent the earliest changes of RHD or are merely variations of normal heart anatomy.

At present, minor non-diagnostic changes of heart morphology are the commonest silent findings when echocardiography is undertaken in otherwise well children. Whether such changes indicate a large burden of minor and undiagnosed RHD that would benefit from secondary prophylaxis or incidental normal variants will be essential in providing an evidence-based rationale for echocardiographic screening in populations at elevated risk of RHD.

Finally this project will continue to support the development of research capacity in northern and remote Australia and of Aboriginal and Torres Strait Islander people. It will enable us to ask and answer health-related questions which are relevant and a priority in informing the response to addressing the disparity in disease burden and health outcome between Indigenous and non-Indigenous Australians.

## Abbreviations

AMVL: Anterior mitral valve leaflet; AR: Aortic regurgitation; ARF: Acute rheumatic fever; ECG: Electrocardiograph; GAS: Group A Streptococcus; gECHO: Getting Every Child’s Heart Okay study; MR: Mitral regurgitation; MV: Mitral valve; PLAX: Parasternal long axis; PMVL: Posterior mitral valve leaflet; PSAX: Parasternal short axis; RHD: Rheumatic heart disease; RhFFUS: Rheumatic Fever Follow-Up Study; WHF: World Heart Federation.

## Competing interests

The author(s) declare that they have no competing interest.

## Authors’ contributions

MR is a researcher and contributed to protocol development and drafted this manuscript. DA is an investigator and contributed to the study design and protocol development. AW is an investigator and contributed to the study design and protocol development. YH is a project manager and contributed to protocol development. AB is an investigator and contributed to the study design and protocol development. JC is an investigator and contributed to the study design and protocol development. GM is an investigator and contributed to the study design, protocol development, and drafting of this manuscript. All authors critically reviewed this manuscript and approved the final protocol.

## Pre-publication history

The pre-publication history for this paper can be accessed here:

http://www.biomedcentral.com/1471-2261/12/111/prepub
